# Support Vector Regression Approach to Predict the Design Space for the Extraction Process of *Pueraria lobata*

**DOI:** 10.3390/molecules23102405

**Published:** 2018-09-20

**Authors:** Yaqi Wang, Yuanzhen Yang, Jiaojiao Jiao, Zhenfeng Wu, Ming Yang

**Affiliations:** 1 College of Pharmacy, Chengdu University of Traditional Chinese Medicine, Chengdu 610072, China; wangyaqi_3@163.com (Y.W.); jqiao6@163.com (J.J.); 2 Key Laboratory of Modern Preparation of Traditional Chinese Medicine, Ministry of Education, Jiangxi University of Traditional Chinese Medicine, Nanchang 330004, China; yangyuanzhen666@163.com (Y.Y.); zfwu527@163.com (Z.W.)

**Keywords:** *Pueraria lobata*, SVR, QPM, extraction process, QbD, design space

## Abstract

A support vector regression (SVR) method was introduced to improve the robustness and predictability of the design space in the implementation of quality by design (QbD), taking the extraction process of *Pueraria lobata* as a case study. In this paper, extraction time, number of extraction cycles, and liquid–solid ratio were identified as critical process parameters (CPPs), and the yield of puerarin, total isoflavonoids, and extracta sicca were the critical quality attributes (CQAs). Models between CQAs and CPPs were constructed using both a conventional quadratic polynomial model (QPM) and the SVR algorithm. The results of the two models indicated that the SVR model had better performance, with a higher *R*^2^ and lower root-mean-square error (*RMSE*) and mean absolute deviation (*MAD*) than those of the QPM. Furthermore, the design space was predicted using a grid search technique. The operational range was extraction time, 24–51 min; number of extraction cycles, 3; and liquid–solid ratio, 14–18 mL/g. This study is the first reported work optimizing the design space of the extraction process of *P. lobata* based on an SVR model. SVR modeling, with its better prediction accuracy and generalization ability, could be a reliable tool for predicting the design space and shows great potential for the quality control of QbD.

## 1. Introduction

Radix puerariae (RP) is the root of *Pueraria lobata*, known as ‘Gegen’ in Chinese. RP was one of the earliest herbal resources used for food and medicine: it was firstly documented in “Shen Nong’s Herbal Classic” and classified as middle grade for the prevention and treatment of fever, diabetes, diarrhea, and cardiovascular and cerebrovascular diseases. Furthermore, modern studies have shown that RP extract exhibits potential bioactivity for the treatment of several immune disorders, such as atopic dermatitis [1], osteoporosis [2], and Alzheimer’s disease [3]. Isoflavonoids are believed to be the major active components in RP. Puerarin is the major and most important component in RP with extensive pharmacological activities such as hepatoprotection [4], anti-atherogenic effects [5], and anti-cancer effects [6]. The content of puerarin (≥2.4%) is regarded as the quality indicator of RP according to the Pharmacopeia of the People’s Republic of China.

Extraction is a key operation process in the manufacturing of health food, dietary supplements, and medicine, especially for traditional Chinese medicine (TCM). The quality by design (QbD) concept has been applied in the pharmaceutical field as a well-established tool for both formulation and manufacturing process development. According to ICH guideline Q8, QbD is implemented with several steps, including risk analysis, diagnosis of potential critical quality attributes (CQAs) and process parameters (CPPs), construction of mathematical models, optimization of the design space, selection of a control strategy, and continual improvement in the product lifecycle [7,8]. Among these steps, design space, as a reliable operation range, has gained increasing attention in the food and drug industries. According to the definition of design space, the quality is guaranteed at any combination of independent variables (process parameters) within the space. Consequently, the design space is regarded as a zone of robustness, as no significant fluctuations should be observed within the space.

Design space is established on the basis of a sound understanding of the effect of the interaction of CQAs and CPPs on the quality of the product. Therefore, statistical and multivariate analysis models are essential in the implementation of QbD. The predictive ability of the mathematical model shows the robustness and predictability of the design space. Furthermore, a well-fitted model helps us not only to gain a clear understanding of the connection and the intrinsic regular pattern between CPPs and CQAs, but also to gain regulatory flexibility. Thus, constructing a reliable model is first and foremost. The robustness of the model directly influences the batch-to-batch consistency of the quality of products.

Response surface methodology (RSM), due to its data visualization and handling ability, has become the most widely used method to express multidimensional relationships between CQAs and CPPs. The quadratic polynomial model (QPM) is the most common algorithm for response surface optimization. Design of experiment (DoE) analysis techniques such as analysis of variance and fitted regression models are used frequently. A multivariate knowledge space may be delineated to find regions of risk or optimal performance, which are often graphically illustrated by figures and known as response surfaces. The literature has shown that the QPM has good performance when used for relatively simple and linear cases [9,10]. The QPM also has some well-known limitations that may affect the prediction accuracy [11]. The multidimensional relationships observed in the pharmaceutical area are often complex and nonlinear. The predictions of models based on the linear regression algorithm exhibit poor estimation [12]. Therefore, the QPM may not be the most applicable algorithm for accurate prediction of the design space of QbD.

Support vector regression (SVR) is a promising kernel-based machine learning algorithm developed by Vapnik and Cortes [13]. The SVR approach can optimize complex nonlinear problems by using an exclusive objective function that minimizes the structural risk of the model. The introduction of the kernel function allows nonlinear problems to be linearly solved in a higher dimension compared with its original dimensional feature space. Thus, SVR has a global optimum and exhibits excellent prediction accuracy. Considering its remarkable generalization performance, SVR has attracted particular attention and been extensively used in applications including atmospheric science prediction [14], drug design [15], credit rating analysis [16], protein structure and function prediction [17], and metabolomics [18]. In most of these cases, the performance of the SVR model is better than that of traditional machine learning approaches. To our knowledge, few studies of the design space model have been developed based on the SVR model.

The aim of this present study was to explore the practicability of using an SVR model for predicting a design space. For the RP extraction process, there are several analytical methods based on the QPM for response surface optimization [19]. A single algorithm may not be credible enough for model development. To the best our knowledge, there is no study in the literature comparing the QPM and the SVR algorithm for the extraction process of RP. Thus, in this paper, the RP extraction process was optimized as a case study. The extraction time, number of extraction cycles, and liquid–solid ratio were identified as CPPs, and the yield of puerarin, total isoflavonoids, and extracta sicca were the CQAs. Models between CQAs and CPPs were constructed using both the QPM and the SVR algorithm based on the Box–Behnken design. The performance of the two models was analyzed and compared. Then, the design space was calculated and optimized using a grid search technique. This is the first study on optimization of the design space of the extraction process of RP using the SVR algorithm.

## 2. Results

### 2.1. Box–Behnken Design 

Box–Behnken design is one of the most commonly used DoEs for RSM. Three influential factors (independent variables) for the extraction process of RP were investigated, including extraction time (*X*_1_, min), extraction cycles (*X*_2_, cycles), and liquid–solid ratio (*X*_3_, mL/g). Seventeen experimental runs were arranged using Box–Behnken design. Their experimental results were considered as dependent variables. In this work, *Y*_1_, *Y*_2_, and *Y*_3_ represented the yields of puerarin (%), extracta sicca (%), and total isoflavonoids (%), respectively. The entire dataset obtained using Box–Behnken design was considered as the training set and adopted to establish the QPM and SVR fitted models. To further evaluate the performance of the two fitted models, cross-validation and external validation approaches were adopted. Four sets of external validation values—random combinations of independent variables along with experimental responses—were used as the test set to evaluate the quality of the fitted models. The experimental datasets are shown in Table 1.

### 2.2. QPM Analysis

A five-fold cross-validation method was used for training and evaluation of both the QPM and the SVR model. The regression coefficient and parameters of QPM and SVR are listed in Table 2. All the fitted *R*^2^ (training set) values of QPM were more than 0.96, which shows that more than 96% of the variation could be explained by QPM. For the yields of puerarin (*Y*_1_) and extracta sicca (*Y*_2_), the predicted *R*^2^ (test set) values were higher than 0.90, which shows that 90% of the variation could be predicted by QPM. The cross-validation *R*^2^ values were above 0.80. The closer the cross-validation *R*^2^ is to 1, the better the generalization capability of the statistical model. The regression coefficients and *p*-values of the constructed QPM equation are presented in Table 3. For the yields of puerarin (*Y*_1_) and extracta sicca (*Y*_2_), the effects of extraction time (*X*_1_), extraction cycles (*X*_2_), and liquid–solid ratio (*X*_3_) were all significant model terms. For the yield of total isoflavonoids (*Y*_3_), there was no significant model term, whereas the fitted *R*^2^ (training set) of the QPM was more than 0.96. This phenomenon may be caused by the combined effect of each model term playing a greater role than any single model term.

### 2.3. SVR Analysis

#### 2.3.1. Parameter Optimization for SVR

Due to the good general performance and the small number of parameters to be adjusted, the Gaussian radial basis function (RBF) was employed as the kernel function in this study. Thus, the SVR performance depends on the combination of three parameters: the capacity parameter *C*, the kernel function parameter *ε*, and the kernel parameter *σ*.

In the SVR formulation, the capacity parameter *C* represents the tradeoff between the margin maximization and the training error minimization. If the *C* value is too high or too low, the algorithm may over- or under-fit the training set. The kernel function parameter *ε* signifies the width of the *ε*-insensitive zone used to fit the training data. Moreover, the corresponding kernel parameter *σ* strongly influences the number of support vectors. Thus, the combination of the three parameters controls the accuracy and generalization performance of the regression estimate.

**Figure 1:**
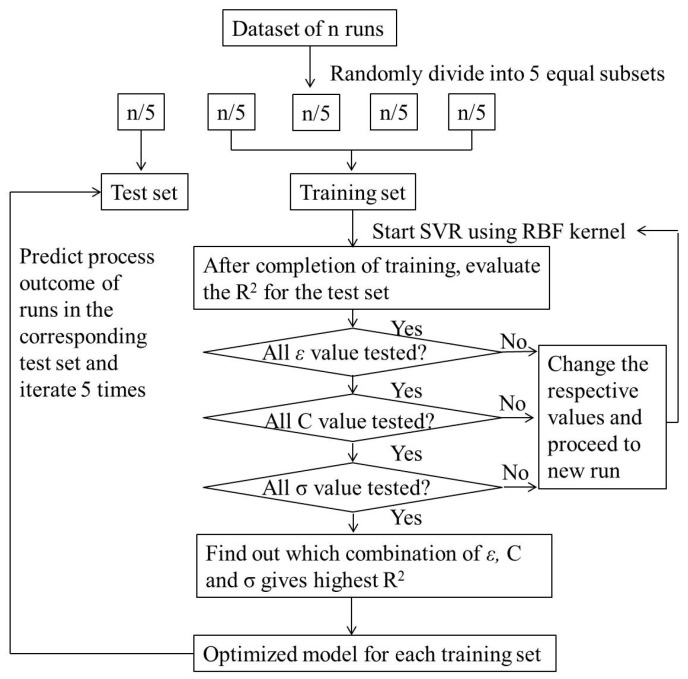
Schematic for SVR algorithm implementation.

To choose the parameters of the model, this paper adopted the method of cross-validation based on a grid search avoiding blindness and randomness. The key is to find out which combination of the three parameters gives the highest prediction accuracy. The procedure for optimizing the parameters of SVR is as follows (schematic shown in Figure 1):

Step 1: Start SVR using an RBF kernel and find the *R*^2^, *RMSE*, and *MAD* for the run with the test set.

Step 2: Repeat the procedure by varying *ε* from 2^−15^ to 2^−1^ (typically 15 values) and find the *R*^2^, *RMSE*, and *MAD* for each run (15 runs).

Step 3: Repeat the procedure by varying *C* (capacity control) from 2^6^ to 2 (typical six values: 2^6^, 2^5^, 2^4^, 2^3^, 2^2^, 2) and find the *R*^2^, *RMSE*, and *MAD* for each run (15 × 6 runs).

Step 4: Repeat the procedure by varying *σ* from 2^−15^ to 2^−1^ (typically 15 values) and find the *R*^2^, *RMSE*, and *MAD* for each run (15 × 6 × 15 runs).

Step 5: Steps 1–4 are repeated for the development of each model of *Y*_1_, *Y*_2_, and *Y*_3_.

The optimized combination of the three parameters for the SVR model is shown in Table 4.

#### 2.3.2. Evaluation of Models 

The performance of the two models was mainly evaluated using two validation techniques: cross-validation and test set. The parameters of the QPM and the SVR model are listed in Table 2. Although the *R*^2^ values of the training set were similar between QPM and SVR, for the test set and cross-validation group, the *R*^2^ values of the SVR model were higher than those of the QPM. Furthermore, the *RMSE* and *MAD* values of SVR for the test set and cross-validation were lower than those of QPM. The high *R*^2^ and low *RMSE* and *MAD* indicate the good prediction and generalization performance of the SVR model.

**Figure 2:**
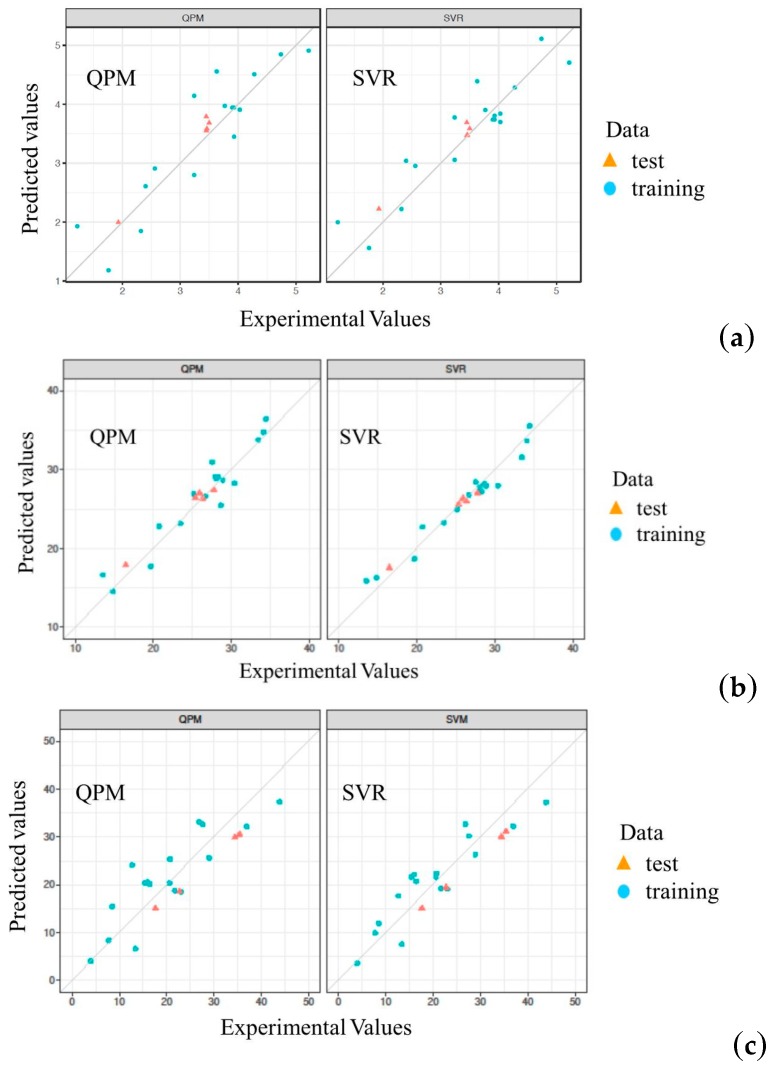
Comparison of predicted and experimental values for QPM and SVR. (**a**) *Y*_1_; (**b**) *Y*_2_; (**c**) *Y*_3_.

Figure 2 depicts a comparison between predicted and experimental values for the training and test sets with QPM and SVR. Obviously, the values predicted by SVR are more closely matched with the experimental runs than are those by QPM. Hence, SVR is superior to QPM for predicting the yield value of the extraction process of RP.

### 2.4. Design Space

**Figure 3:**
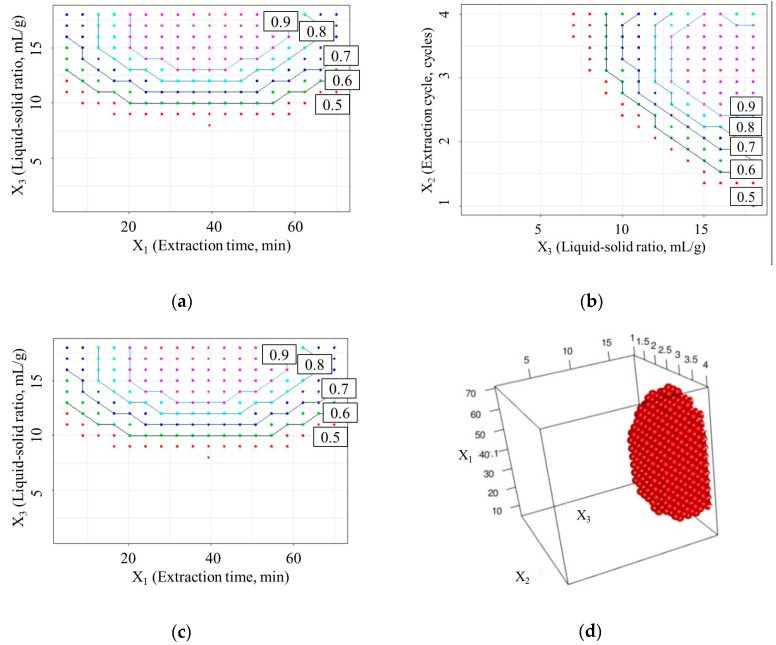
Design space and contour plots: (**a**) extraction cycle (*X*_2_) fixed at 3 cycles; (**b**) extraction time (*X*_1_) fixed at 35 min; (**c**) liquid-to-solid ratio (*X*_3_) fixed at 9 mL/g; (**d**) four-dimensional operation space (D > 0.9).

In this study, the aim of the optimization was to obtain the design space (operating range) which could provide the maximum yield value of active components and minimum of extracta sicca. Figure 3 shows the design space of the optimized yield value (D) based on the SVR model. It was observed that the yield value of D increased with the extraction time (*X*_1_) up to 20 min, and then declined for *X*_1_ greater than 55 min. This phenomenon may be caused by the increased extraction time accelerating the mass transfer. When the extraction time continuously increases, the extracta sicca grows faster than the active components’ dissolution rate. When the extraction time reaches 55 min, the D value begins to decline. However, the number of extraction cycles (*X*_2_) and liquid–solid ratio (*X*_3_) both had a positive effect on the extraction efficiency. Increased *X*_2_ and *X*_3_ enhanced the extraction efficiency greatly, which shows that the concentration gradient of the solvent plays a critical role in the RP extraction process. However, a continuously increasing number of extraction cycles and liquid-solid ratio may result in a heavy load for the subsequent concentration and drying process.

For a maximum yield value of active components and minimum extracta sicca, a D value of 0.9 was set as the lower limit. The design space was calculated using a grid search method. The four-dimensional design space is shown in Figure 3d. The design space is an irregular polygon, and the operational range for the extraction process is *X*_1_, 24–51 min; *X*_2_, 3 cycles; and *X*_3_, 14–18 mL/g.

The predicted operating range was validated using an external validation approach which consisted of a combination of CPPs that never occurred in the calibration set. Verification results are shown in Table 5. All the optimized D values were higher than 0.9, in perfect agreement with the predicted values. The good correlation between these results indicates that the design space was reliable in predicting the operating range.

## 3. Materials and Methods

### 3.1. Materials

RP was collected from Pingwu, Sichuan Province (China). Reference samples of puerarin and rutin were purchased from the National Institute for the Control of Pharmaceuticals and Biological Products (Beijing, China). Acetonitrile (chromatographic grade) and methanol (chromatographic grade) were obtained from Merck (Darmstadt, Germany). Deionized water was produced using a Milli-Q academic water purification system (Millipore, Bedford, MA, USA). Sodium nitrite (analytical grade), aluminum nitrate (analytical grade), and sodium hydroxide (analytical grade) were purchased from Sigma (Saint Louis, MO, USA).

### 3.2. Apparatus

An Agilent 1200 HPLC equipped with a variable-wavelength ultraviolet detector (Agilent Technologies, Santa Clara, CA, USA) was used for HPLC analysis. A Phenomenex reversed-phase Gemini C_18_ column (250 × 4.6 mm, 5 μm) and a Phenomenex C_18_ guard column (Phenomenex, Torrance, CA, USA) were used for chromatographic analysis. A Shimadzu UV-2550 UV–vis Spectrophotometer (Shimadzu, Kyoto, Japan) was used for UV analysis.

### 3.3. Procedures

According to the Chinese pharmacopoeia, heat reflux extraction was used to extract active components from RP using water as the solvent. Before extraction, RP samples were oven-dried at 60 °C for 12 h. Each sample was cut into half-inch cubes. A 100 g sample was placed in a round-bottom flask and soaked with 5–15 mL/g (500–1500 mL) of water for 30 min. Then, heat reflux extraction was performed for 1–3 cycles of 10–60 min each according to the experimental design. After extraction, the mixture was filtered with gauze. The supernatant was subjected to HPLC, UV analysis, and weight measurement of extracta sicca. Dried extracta sicca were obtained by firstly evaporating in a 60 °C water bath and then drying in a 105 °C oven for 6 h. The yields of puerarin (*Y*_1_), extracta sicca (*Y*_2_), and total isoflavonoids (*Y*_3_) were calculated using the equation *Y* = (mass of analyte/mass of plant material) × 100%. Each experiment was conducted in triplicate and the average yield value was used for statistical analysis.

### 3.4. HPLC Analysis

The yield of puerarin (*Y*_1_) extracted from RP was analyzed using an HPLC approach. The detection wavelength was 254 nm. The gradient elution of the mobile phase contained (A) acetonitrile and (B) water with 0.1% formic acid. Gradient procedures were as follows: 0–25 min, 11% A; 25–30 min, 11–25% A; 30–40 min, 25–40% A. The flow rate was 1.0 mL/min and the injection volume was 10 μL. Column temperature was maintained at 30 °C. The linear range of puerarin was 10–1000 μg/mL, *Y* = 28.981*X* + 112.29 (*R*^2^ = 0.9997). The limits of detection (LOD) and quantification (LOQ) were 0.6 and 2 μg/mL, respectively.

### 3.5. UV Analysis

The total quantity of isoflavonoids (*Y*_3_) extracted from RP was measured using an aluminum nitrate colorimetric method described by Saeed [20]. Briefly, in a 10 mL test tube, 1 mL of the extract, 0.4 mL 5% sodium nitrite, and 0.4 mL 10% aluminum nitrate were mixed. After 6 min, 4 mL 4% sodium hydroxide and 4.2 mL 75% ethanol were added, mixed well, and left to stand for 15 min. Then, the absorbance of the solution was measured against a prepared reagent blank at 510 nm using a UV–vis spectrophotometer. The standard curve for total isoflavonoids was found using a rutin standard solution (0–80 mg/L) under the same procedure described above. The content of total isoflavonoids was expressed as milligrams of rutin equivalent per gram of dried sample. All the samples were analyzed in duplicate.

### 3.6. Establishment of Models

#### 3.6.1. QPM

A second-order response function was applied to establish a mathematical model that relates the response measured to the independent variables

(1) $$Y=b_{0}+\sum_{i=1}b_{i}X_{i}+{\sum_{i=1}b_{ii}X_{i}}^{2}+\sum_{i,j=1}b_{ij}X_{ij}$$

where *Y* is the response, *b*_0_ is a constant, *b_i_* is the linear coefficient, *b_ii_* is the quadratic coefficient, *b_ij_* is the two-factor interaction coefficient, and *X_i_* and *X_j_* are the process parameters.

The quality of the fitted models was expressed using the coefficient of determination (*R*^2^), *RMSE*, and *MAD*.

#### 3.6.2. SVR

By introducing the kernel function, the original input was mapped into the feature space. The ultimate mathematical form of the kernel-based SVR is shown in Equation (2)

(2) $$f(x)=\sum_{i=1}^{N}\left(\alpha_{i}^{*}-\alpha_{i}^{}\right)K(x_{i},x)+b_{}$$

(3) $$b_{}=\{\begin{array}{c} y_{i}-f{\left(x_{i}\right)}_{b=0}-\varepsilon \begin{array}{ccc} & & \begin{array}{cc} & For\begin{array}{cc} & \alpha_{i}^{}\in (0,C) \end{array} \end{array} \end{array}\\ y_{i}-f{\left(x_{i}\right)}_{b=0}+\varepsilon \begin{array}{ccc} & & \begin{array}{cc} & For\begin{array}{cc} & \alpha_{i}^{*}\in (0,C) \end{array} \end{array} \end{array} \end{array}$$

where *α_i_* and α_i_^*^ are the optimized Lagrange multipliers, *K*(*x_i_*, *x*) denotes the kernel function describing the dot product in the feature space, *b* is the bias parameter, and *x_i_* and *y_i_* denote the *i*th support vector and the corresponding target output, respectively. The coefficients *α* and α^*^ have an intuitive interpretation as forces pushing and pulling the regression estimate *f*(*x*) towards the measurements *y*.

The kernel function is defined in terms of the dot product of the mapping function as given by

(4) $$K(x_{i},x_{j})=\langle \phi (x_{i}),\phi (x_{j})\rangle$$

where *Ф*(*x*) is the high-dimensional feature space being nonlinearly mapped to from the input space *x*. There exist several choices for the kernel function *K*, including linear, polynomial, splines, and radial basis functions. With respect to the support vector regression, the function which is broadly employed is the Gaussian RBF.

(5) $$K(x_{i},x_{j})=\exp(\frac{{\left\|x_{i}-x_{j}\right\|}^{2}}{2\sigma^{2}})$$

### 3.7. Optimization

A function was used to optimize the three CQAs simultaneously: D = *Y*_1_ + *Y*_3_ − *Y*_2_. D is the optimized yield value of *Y*_1_, *Y*_2_, and *Y*_3_. All the CQA data were normalized using the equation *Y*’ = (*Y − Y*_min_)/(*Y*_max_ − *Y*_min_). In our study, the goal of optimization was to the maximize yield value of active components and minimize the extracta sicca. Extraction time *X*_1_ (0–70 min), number of extraction cycles *X*_2_ (1–4 cycles), and liquid–solid ratio *X*_3_ (0–18 mL/g) were chosen as the recognition space. A grid search method was used to seek the design space.

The software RStudio (v1.1.456, RStudio, Boston, MA, USA) and R v3.5 were used to construct the QPM and SVR model, and to perform the grid search method.

## 4. Conclusions

In this study, the SVR algorithm was used to improve model performance for finding the design space of the RP extraction process. The results indicate that the SVR model performs better, with higher *R*^2^ and lower *RMSE* and *MAD* than those of QPM. Furthermore, verification results were in perfect agreement with the predicted values, which indicated that the SVR model was reliable in predicting the operating range for the extraction process of RP. This study is the first reported work optimizing the design space of the RP extraction process based on an SVR model. SVR modeling has better prediction accuracy and generalization ability, and shows great potential for the quality control of QbD as a reliable tool for predicting the design space.

## Figures and Tables

**Table 1:** Results of training and test set.

No.	Factors			Response Variables		
	*X*_1_ (min)	*X*_2_ (cycles)	*X*_3_ (mL/g)	*Y*_1_ (%)	*Y*_2_ (%)	*Y*_3_ (%)
Training set						
1	35	3	5	3.93	28.64	16.00
2	35	3	15	5.22	34.11	43.86
3	10	3	10	3.63	27.53	28.95
4	35	2	10	4.03	28.06	16.40
5	60	3	10	4.74	34.44	26.82
6	35	2	10	3.90	27.97	20.60
7	35	1	5	1.22	13.52	39.20
8	10	2	15	3.24	25.18	27.57
9	60	2	5	3.24	26.67	13.37
10	10	1	10	1.76	14.82	7.73
11	35	2	10	3.93	28.25	15.41
12	60	1	10	2.40	23.47	12.66
13	35	2	10	4.03	30.40	23.03
14	60	2	15	4.28	33.46	36.94
15	35	2	10	3.77	28.90	21.69
16	10	2	5	2.32	19.67	8.46
17	35	1	15	2.56	20.74	20.70
Test set						
1	25	2	10	3.50	25.92	22.70
2	30	2	8	3.45	25.37	17.62
3	15	2	15	3.46	26.35	34.41
4	20	2	15	3.45	27.78	35.40

**Table 2:** Statistical parameters of the quadratic polynomial model (QPM) and the support vector regression (SVR) model.

		QPM			SVR		
		*R* ^2^	*RMSE*	*MAD*	*R* ^2^	*RMSE*	*MAD*
***Y*_1_**	Training set	0.985	0.127	0.111	0.983	0.132	0.077
	Test set	0.903	0.191	0.164	0.918	0.175	0.133
	Cross-validation	0.802	0.457	0.366	0.846	0.403	0.329
***Y*_2_**	Training set	0.988	0.641	0.514	0.982	0.789	0.596
	Test set	0.944	0.946	0.797	0.975	0.636	0.559
	Cross-validation	0.908	1.795	1.429	0.954	1.272	1.031
***Y*_3_**	Training set	0.964	1.906	1.56	0.961	2.005	1.646
	Test set	0.706	4.12	4.02	0.765	3.683	3.606
	Cross-validation	0.724	5.311	4.567	0.821	4.281	3.834

**Table 3:** Coefficients of the constructed QPM equation.

	QPM										
		C_0_	*X*_1_^2^	*X*_2_^2^	*X*_3_^2^	*X*_1_ *X*_2_	*X*_1_ *X*_3_	*X*_2_ *X*_3_	*X*_1_	*X*_2_	*X*_3_
***Y*_1_**	Regression coefficient	0.751	−1.524	−1.674	−1.124	2.157	3.859	2.237	0.470	0.120	−0.050
	*p*-value	0.014 *	0.006 *	0.003 *	0.023 *	0.275	0.771	0.903	0.003 *	0.001 *	0.003 *
***Y*_2_**	Regression coefficient	9.371	−3.317	−11.287	−6.567	11.257	26.075	13.050	−1.740	1.280	−1.750
	*p*-value	0.001 *	0.132	0.001 *	0.012 *	0.413	0.542	0.41	0.003 *	0.001 *	0.001 *
***Y*_3_**	Regression coefficient	1.402	0.158	−1.702	8.478	5.412	17.347	5.582	−7.060	4.460	11.080
	*p*-value	0.698	0.979	0.777	0.186	0.273	0.477	0.104	0.491	0.053	0.478

* Significant at the 0.05 level.

**Table 4:** Optimized parameters for the SVR model.

	SVR		
	*σ*	C	*ε*
*Y*_1_	2^−4^	2^2^	2^−6^
*Y*_2_	2^−^^5^	2^4^	2^−^^3^
*Y*_3_	2^−4^	2^5^	2^−^^3^

**Table 5:** Responses for verification experiments.

No.	Factors			Predicted D Value	Experimental D Value
	*X*_1_ (min)	*X*_2_ (cycles)	*X*_3_ (mL/g)		
1	35	3	14	0.98	0.99
2	40	3	15	1.03	1.01
